# A systematic review of interventions to increase breast and cervical cancer screening uptake among Asian women

**DOI:** 10.1186/1471-2458-12-413

**Published:** 2012-06-07

**Authors:** Mingshan Lu, Sabina Moritz, Diane Lorenzetti, Lindsay Sykes, Sharon Straus, Hude Quan

**Affiliations:** 1 Departments of Economics and Community Health Sciences, University of Calgary, Calgary, AB, Canada; 2 Canadian Institute of Natural and Integrative Medicine, Calgary, AB, Canada; 3 Department of Community Health Sciences and Centre for Health and Policy Studies, University of Calgary, Calgary, AB, Canada, T2N 1 N4; 4 Department of Health Policy, Management, & Evaluation, University of Toronto, Toronto, ON, Canada

## Abstract

**Background:**

The Asian population is one of the fastest growing ethnic minority groups in western countries. However, cancer screening uptake is consistently lower in this group than in the native-born populations. As a first step towards developing an effective cancer screening intervention program targeting Asian women, we conducted a comprehensive systematic review, without geographic, language or date limitations, to update current knowledge on the effectiveness of existing intervention strategies to enhance breast and cervical screening uptake in Asian women.

**Methods:**

This study systematically reviewed studies published as of January 2010 to synthesize knowledge about effectiveness of cancer screening interventions targeting Asian women. Fifteen multidisciplinary peer-reviewed and grey literature databases were searched to identify relevant studies.

**Results:**

The results of our systematic review were reported in accordance with the PRISMA Statement. Of 37 selected intervention studies, only 18 studies included valid outcome measures (i.e. self-reported or recorded receipt of mammograms or Pap smear). 11 of the 18 intervention studies with valid outcome measures used multiple intervention strategies to target individuals in a specific Asian ethnic group. This observed pattern of intervention design supports the hypothesis that employing a combination of multiple strategies is more likely to be successful than single interventions. The effectiveness of community-based or workplace-based group education programs increases when additional supports, such as assistance in scheduling/attending screening and mobile screening services are provided. Combining cultural awareness training for health care professionals with outreach workers who can help healthcare professionals overcome language and cultural barriers is likely to improve cancer screening uptake. Media campaigns and mailed culturally sensitive print materials alone may be ineffective in increasing screening uptake. Intervention effectiveness appears to vary with ethnic population, methods of program delivery, and study setting.

**Conclusions:**

Despite some limitations, our review has demonstrated that the effectiveness of existing interventions to promote breast and cervical cancer screening uptake in Asian women may hinge on a variety of factors, such as type of intervention and study population characteristics. While some studies demonstrated the effectiveness of certain intervention programs, the cost effectiveness and long-term sustainability of these programs remain questionable. When adopting an intervention program, it is important to consider the impacts of social-and cultural factors specific to the Asian population on cancer screening uptake. Future research is needed to develop new interventions and tools, and adopt vigorous study design and evaluation methodologies to increase cancer screening among Asian women to promote population health and health equity.

## Background

Breast cancer continues to be the most common cancer and the second leading cause of cancer death for women in Western countries [1,2]. In 2011, approximately 230,480 females in the US were diagnosed with breast cancer. The death toll for that same year was estimated at 39,520 deaths [3]. Cervical cancer is the second most common cancer among women, with an estimated 12,710 new cases diagnosed in the US in 2011 and 4,290 reported deaths [4]. Some 80% of cervical cancers occur in developing countries [5]. In addition, mortality rate of breast cancer and cervical cancer among Asian women is similar to that of Caucasian women. In 2002–2006, the age-adjusted death rate of breast cancer among Asian women in the U.S. was 81.6 per 100,000 (vs. 123.5 for Caucasians); and 2.2 per 100,000 (vs. 2.2 for Caucasians) for cervical cancer [4].

Regular mammogram screening and Pap smear testing have been utilized to detect breast and cervical cancer at early stages, and have been shown to be effective in reducing breast and cervical cancer deaths [6-9]. The success and effectiveness of an organized cancer screening program is largely dependent on obtaining high participation rates through effective recruitment and retention strategies. However, breast and cervical cancer screening rates are consistently low among Asian women, both in Asian and Western countries. In the United States, Asian Americans and Pacific Islanders have the lowest breast and cervical screening rates among all ethnic groups [3,4]. A study published in 2000 reported that compared with 21% of white women in the same sample, 30% of Asian women (26% Chinese, 21% Japanese, 28% Filipino, 50% Korean, and 68% Asian Indians) had never had a mammogram [10]. Similarly, 21% Asian women (28% Chinese, 8% Japanese, 15% Filipino, 25% Korean, 36% Vietnamese, and 26% Asian Indians) never had a Pap test compared with only 5% of white women in the sample [10]. Similar low breast and cervical cancer screening rates for Asian women have been reported in countries such as Canada [11], UK [12-14] and Australia [15]. For Asian women, barriers to cancer screening utilization include cognitive barriers (knowledge about screening, understanding the purpose of the test, or benefits of testing for early detection), emotional barriers (fear/social stigma), economic barriers (time, taking time off work, insurance coverage), logistic barriers (lack of consistent physician, limited office hours, childcare, transportation, waiting times, language barriers) and social barriers (support of family and friends, support within the physician’s office) [16].

According to the most recent census data, 12.5% of the American population and 19.8% of the Canadian population are foreign-born [17,18]. Of these, 27.7% of US and 58.3% of Canadian immigrants were born in Asia. It has been predicted that the Asian population will continue to be one of the fastest growing ethnic populations in these countries [17,18]. Because of the widely recognized need to ensure equal access to high quality health care services for all citizens, this growth of Asian populations in western countries has garnered the attention of both researchers and policy makers. Given low breast and cervical cancer screening rates among Asian women, it is important to develop interventions to increase cancer screening among Asian women to promote population health and health equity.

Numerous intervention strategies have been studied to promote breast and cervical cancer screening uptake among Asian populations. Han and colleagues completed a meta-analysis of intervention studies in the United States from 2000 until 2008 to promote mammography among ethnic minority women, including Asian women. Access-enhancing interventions (e.g., mobile vans and reduced-cost mammograms) were shown to be most effective, followed by individually directed interventions (e.g., one-on-one counseling, tailored and non-tailored letters and reminders, and telephone counseling). Tailored, theory-based interventions (e.g., providing intervention materials designed with cultural sensitivities to suit the individual characteristics) were shown to be more effective than non-tailored interventions. Interventions involving community members as a way to address cultural sensitivities were shown to have increased effectiveness compared with other methods such as providing culturally matched materials [19]. Masi and colleagues conducted a systematic review of publications in English from 1986 through 2005 to determine the effectiveness of interventions in the United States to improve breast cancer screening among ethnic minority women. They found that culturally tailored interventions and those that addressed financial or logistical barriers were more successful than reminder-based interventions [20]. Legler and colleagues conducted a meta-analysis of international intervention studies published in English from 1984 to 2000 to promote mammography among women with historically lower rates of screening than the general population. They found that the most effective intervention programs involved a combination of access-enhancing and individual-directed strategies [21]. These previously published reviews on interventions to enhance breast and cervical cancer screening examined either intervention programs targeting all minorities without specifying individual groups, or only selected Asian groups in North America [19-21].

As a first step towards developing an effective cancer screening intervention program targeting Asian women, we conducted a comprehensive systematic review, without geographic, language or date limitations, to update current knowledge on the effectiveness of existing intervention strategies to enhance breast and cervical screening uptake in these populations.

## Methods

### Data sources and searches

A total of 15 interdisciplinary peer-reviewed and grey literature databases were searched including MEDLINE, EMBASE, Cochrane Database of Systematic Reviews, Cochrane CENTRAL Register of Controlled Trials, CINAHL, CancerLit, DARE Database of Reviews of Effects, PsycINFO, ABI Inform, ERIC, Social Sciences Abstracts, Sociological Abstracts, Health Technology Assessment Database (University of York), Proquest Dissertations and Theses, and KUUC Knowledge Utilization Database (University of Laval). We implemented a search strategy which combined, using the Boolean Operator AND, text-words and subject headings (MeSH or equivalent) representing the three concepts relevant to our research question: Concept One: breast cancer screening or breast neoplasms screening or cervical cancer screening or cervical neoplasms screening or mammogram or pap smear or pap test or papanicolaou test or vaginal smear; Concept Two: Asian or Chinese or ethnic or Indian or minority or minorities or Vietnamese; Concept Three: campaign or educat* or intervention* or program* or promote or promoting or promotion or uptake.

We searched for studies published as of January 2010. No language limits were applied. Reference lists of included studies were also scanned to identify additional relevant papers. A complete search strategy is available from the authors.

### Study selection

Inclusion criteria for articles were: 1) the study provided an evaluation or description of a breast and cervical cancer screening program/educational intervention for Asian women and 2) eligible participants were Asians populations in either home or adopted countries. We excluded studies that 1) evaluated breast self-examination, self-swabs for Pap testing, or visual inspection with acetic acid or 2) focused on patients with existing breast or cervical cancer. We did not impose any age limitation on the target population. The reason is that recommendation of cancer screening test varies by guidelines and in some countries, there is even no guideline.

### Synthesis of results and quality assessment

The results of our systematic review are reported in accordance with the PRISMA Statement (http://www.prisma-statement.org). All six authors were divided into three groups, with two reviewers in each group; abstracts were then assigned to the three groups for review. The two reviewers in each group independently reviewed all abstracts for inclusion, applying the criteria outlined above. In cases of doubt based on abstracts, the articles were included for full text articles review. Two investigators then independently reviewed all full text articles to confirm whether inclusion criteria were met. Although we did conduct pre-testing of inclusion/exclusion criteria, we did not calculate inter-rater reliability. Disagreements were resolved by consensus or reference to a third reviewer. Reviewers were not blinded to study author, institution, or journal.

**Figure 1:**
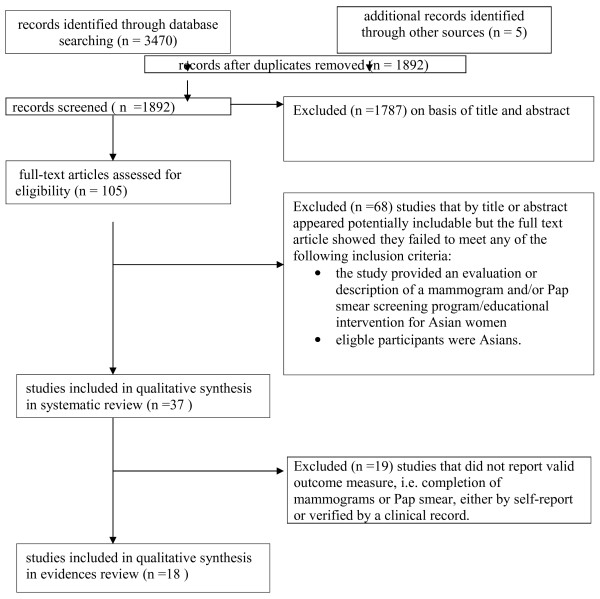
PRISMA flow diagram of studies.

We synthesized the data from our included studies in two ways: first, we presented the study design and intervention strategies for all 37 studies that met inclusion criteria for this review. We believe in the value of describing all interventions that have been implemented to increase breast and cervical cancer screening among Asian women. This information would be particularly useful to those considering implementing an intervention strategy to address cancer screening uptake, even if evidence have not yet been established for all of these strategies. Second, we imposed the quality assessment/outcome measure criteria and reported the evidence on intervention effectiveness for a subset of studies that reported valid outcome measures. This is also clarified in the PRISMA flow diagram of studies (Figure 1).

The 37 studies that met inclusion criteria for the review were found to be very heterogeneous in regard to the intervention methods used and as a result it was not possible to conduct a meta-analysis. The intervention studies were separated into three groups: breast cancer screening, cervical cancer screening, and those studies targeting both breast cancer and cervical cancer screening.

Study designs were classified into randomized control trial (including cluster randomized trial, and randomized controlled crossover trial), non-equivalent control group, or prospective cohort. In order to present reliable evidence on intervention effectiveness, quality assessments were conducted following the criteria described in section 6.4 of the Data Collection Checklist from the Cochrane EPOC guidelines [22]. Studies which did not report valid outcome measures were excluded. Valid outcome measures were defined as completion of mammograms or Pap smear, either by self-report and/or verified by a clinical record. Studies only reporting outcome measures such as increase in knowledge, or willingness/intention of getting screening in the future were not included.

The Jadad scoring system was applied to the assessment of quality of the included randomized control trials (RCTs) [23]. Due to the heterogeneity of the interventions and outcomes, no guideline was deemed adequate to assessing the quality of the observational studies included in this review. As such, we have confined our quality assessment to included RCTs and reported study designs and sample sizes for observational studies.

## Results

As indicated in the PRISMA flow diagram of studies in Figure 1, 3470 records were identified through database searching for a total 1892 unique citations. All citations were screened for inclusion by two authors, using study selection criteria outlined above. Through this process, a total of 105 studies were selected for full text review. Of the 105 abstracts, 37 studies were included in this review (see Tables 1, 2 and 3). Thirteen studies targeted breast cancer screening only, fifteen targeted cervical cancer screening only, and nine focused on both breast cancer and cervical cancer screening. Of these 37 intervention studies, 18 were situated in the US (48.6%); 5 in Taiwan (13.5%), 3 in Thailand (8.1%), 3 in the UK (8.1%), 1 in Canada (2.7%), 1 in Singapore (2.7%), 1 in Australia/Thailand (2.7%), 1 in New Zealand (2.7%), 1 in Hong Kong (2.7%), 1 in India (2.7%), 1 in Malaysia (2.7%), and 1 in the US and Canada (2.7%). The target populations of these interventions included Asian immigrants or women living in their home countries in Asia. The intervention population sample size ranges from 72 to 29,073. Out of the 37 selected intervention studies, 28 (75.7%) were implemented and evaluated within a one to two year period; 6 (16.2%) within a three to five year period; and only 2 (5.4%) with a seven or eight year period.

**Table 1:** Breast cancer screening intervention studies on Asian women

Study*	**Description of Intervention**	**Target Population**	**Sample Size**	**Location**
Australia and Thailand collaborative study 2005 [24]	Culturally Sensitive Print Materials Workplace Based Group Education	Australian and Thai women	114 (Australia); 156 (Thailand)	Three similar bluecollar industries in the Hawkesbury area in New South Wales, Australia and in Chiang Mai, Thailand
Toronto 2005[25]	Culturally Sensitive Print Materials (Mailed)	South Asian immigrant women	72	Toronto, Canada
“Let’s Talk Between Women” 2002 [26]	Church Based Group Education Culturally Sensitive Print Materials Mobile Screening Services	Korean American	147	Los Angeles County, California
Maryland 2002 [27]	Community Based Group Education Culturally Sensitive Print Materials Culturally Sensitive Audiovisual Materials	Korean-American	95 (control); 105 (intervention)	Baltimore Washington Metropolitan, Maryland, US
The Asian Grocery Store-Based Cancer Education Program 2000 [28-34]	Grocery Store Based Group Education	Korean, Vietnamese, Japanese, and Filipino American	Korean: 123 (baseline survey); 93 (follow-up survey); Vietnamese: 275; Japanese: 47; Filipino: 248 (baseline and follow-up surveys); 58 (focus group)	San Diego County, California
“Life is Precious” Project 1999-2002 [35]	Culturally Sensitive Print Materials Culturally Sensitive Audiovisual Materials Community Based Group Education	Hmong women	302 (female)/314 (male)	Three regions (Fresno, Long Beach, and San Diego) in California, US
Los Angeles 1998-2000 [36]	Community Based Group Education Mobile Screening Services	Older Asian American women	49	Los Angeles, California
Alameda 1996-1998 [37]	Culturally Sensitive Print MaterialsFree/Subsidized Screening ServicesMedia Campaigns Screening Education for Health Care ProfessionalsCommunity Based Group Education	Vietnamese-American	384 (intervention); 404 (control)	Alameda County, California
Newham 1995 [38]	Cultural Awareness Training for Health Care Professionals	Indian; Pakistani; Bangladeshi; Chinese	2064	Inner London borough of Newham, UK
Singapore 1994-1996 [39]	Culturally Sensitive Print Materials (Mailed) Home Education Visits	Chinese; Malays; Indians	1500	Singapore
Rochester 1993-1994 [40]	Home Education Visits Screening Reminders/Invitations (Letters) Case Management	Asian	376	Rocherster, US
Oldham 1991 [41]	Home Education Visits	Pakistani and Bangladeshi	527	Oldham, the UK
BCSP (Breast Cancer Screening Program) 1988-1995 [42]	Screening Reminders/Invitations (Letters)	Chinese, Japanese, Vietnamese, and Korean	857 (Asian-American); 1473 (controls)	Washington state, US

* Note: Sometimes there are multiple papers published on one intervention program. In the Study column, we put in the name and period of the intervention program, as well as the references (i.e. “BCSP (Breast Cancer Screening Program) 1988-1995 [42]”). In cases where the intervention programs did not have any name, we put in the site and period of the intervention program, as well as the references (i.e. “Toronto 2005 [25]”).

**Table 2:** Cervical cancer screening intervention studies on Asian women

Study*	**Description of Intervention**	**Target Population**	**Sample Size**	**Location**
Thailand 2006 [43]	Home Education Visits	Thai	304	Khon Kaen, Thailand
Hong Kong 2004 [44]	Cultural Awareness Training for Health Care Professionals Screening Education for Health Care Professionals	Chinese	116	Hong Kong
Pennsylvania 2004 [45]	Assistance in Scheduling/Attending Screening Community Based Group EducationCulturally Sensitive Audiovisual MaterialsFree/Subsidized Screening Services Community Based Group Education	Korean American	102	Pennsylvania, US
Taiwan 2004 [46]	Community Based Group Education	Chinese	N/A	Kaohsiung, Taiwan
Thailand 2003 [47]	Screening Education for Healthcare Professionals	Thai	102 (intervention);103 (control)	Thailand
Taiwan Cervical Screening Program 2002-2004 [48]	Mobile Screening ServicesScreening Reminders/Invitations (Letters) Screening Reminders/Invitations (Telephone Calls)	Chinese	29,073	Taiwan
Lay Health Worker Outreach 2001-2004 [49-51]	Community Based Group Education Media Campaigns	Vietnamese American	400 (2003 evaluation);968 (2006 evaluation)	Santa Clara County, California, US
Seattle 2000-2001 [52]	Assistance in Scheduling/Attending Screening Community Based Group EducationCulturally Sensitive Print Materials Culturally Sensitive Audiovisual MaterialsHome Education Visits InterpretersFree Transportation	Cambodian American	370	Seattle, US
Breast and Cervical Cancer Control Program 1999-2004 [50]	Assistance in Scheduling/Attending Screening Free/Subsidized Screening ServicesHome Education Visits Media Campaigns Screening Education for Health Care ProfessionalsScreening Reminders/Invitations (Letters)	Vietnamese American	1566	Santa Clara County, California
Seattle and Vancouver Trial 1999 [52,53]	Culturally Sensitive Print Materials (Mailed) Culturally Sensitive Audiovisual Materials Home Education Visits	Chinese	482 (2002 evaluation);139 (2007 cost-effectiveness evaluation)	Seattle, Washington, US; and Vancouver, British Columbia, Canada
Taiwan 1999 [54,55]	Assistance in Scheduling/Attending Screening Culturally Sensitive Print MaterialsScreening Reminders/Invitations (Letters)Screening Reminders/Invitations (Telephone Calls)	Chinese	424	Taiwan
Taiwan 1997-1998 [56]	Community Based Group EducationCulturally Sensitive Print Materials (Mailed)	Chinese	333: 66(pre-tested experimental group; 57 (pretested control group); 64 (nonpretested experimental group; 63 (nonpretested control group)	Taipei, Taiwan
India 1996 [57]	Community Based Group Education	Indian	2,864	India
Brisbane 1994 [58]	Media CampaignsScreening Reminders/Invitations (Letters)	Vietnamese	689	Brisbane, Australia
Thailand 1993 [59]	Mobile Screening Services	Thai	1603(1991 survey); 1369(1994 survey)	Thailand
New Zealand 1987-1988 [60]	Culturally Sensitive Audiovisual Materials Culturally Sensitive Print Materials (Mailed ) Home Education Visits	Indian, Pakistani, Bangladeshi	737	Leicester, New Zealand

**Table 3:** Breast and cervical cancer screening intervention studies on Asian women

Study*	**Description of Intervention**	**Target Population**	**Sample Size**	**Location**
The Keelung Community-Based Multiple Disease Screening Programme 1999-2003 [61]	Screening Reminders/Invitations (Letters) Screening Reminders/Invitations (Telephone Calls) Assistance in Scheduling/Attending Screening Media Campaigns	Chinese	N/A	Keelung, Taiwan
Los Angeles 1998-2000 [62]	Community Based Group EducationHome Based Group Education	Filipino American	530	Los Angeles, California
Early Cancer Surveillance Program 1994-1999 [63]	Culturally Sensitive Print MaterialsMedia Campaigns Screening Education for Health Care Professionals	Malays	N/A	Sarawak, Malaysia
Minnesota 1994 [64,65]	Assistance in Scheduling/Attending Screening Community Based Group EducationFemale Physicians Free TransportationFree/Subsidized Screening Services InterpretersScreening Reminders/Invitations (Telephone Calls)	Vietnamese and Cambodian American	90 (Vietnamese); 57 (Cambodian)	Olmsted County, Minnesota, US
Tell a Friend, Alameda 1994-2002 [66,67]	Church-Based Group Education	Korean Americans	818 (1994 survey); 72 (1997 survey); 1084 (2002 survey)	Alameda County, California
	Culturally Sensitive Print Materials Financial Screening IncentivesFree/Subsidized Screening Services Media Campaign Assistance in Scheduling/Attending Screening			
The Breast and Cervical Cancer Intervention Study 1993-1996 [68]	Home Education VisitsScreening Education for Health Care ProfessionalsScreening Followup Community Based Group Education	Chinese	136 (intervention); 135 (control)	San Francisco Bay Area, California
Lay Health Workers Outreach 1992-1996 [69]	Community Based Group Education	Vietnamese Americans	306 (1992); 373 (1996)	San Francisco, California
	Culturally Sensitive Print Materials			
	Media Campaigns			
Media-Led Education Campaign 1992-1994 [70]	Culturally Sensitive Print Materials	Vietnamese American	451 (intervention); 482 (control)	Alameda and Santa Clara Counties (intervention sites), Los Angeles and Orange Counties, US (control sites)
	Media Campaigns			
Bradford 1991-93 [71]	Community Based Group Education	South Asian	670	Bradford, UK

The included studies are extremely diverse in terms of the intervention strategies that were adopted. Most studies used multiple strategies. As described in Table 1, intervention strategies to enhance breast cancer screening included those targeting both patients and health care professionals. Interventions targeting patients included two types: individual-base**d** interventions, including culturally sensitive print or audiovisual materials, home education visits, screening reminders/invitations (letters), case management, mobile screening services, free/subsidized screening services; and, group based interventions including community based, workplace based, church based, grocery store based group education, and media campaigns. Interventions that targeted health care professionals included cultural awareness training and screening education for health care professionals. As described in Table 2, Intervention strategies to enhance cervical cancer screening included assistance in scheduling/attending screening and screening reminders/invitations (telephone calls). Studies presented in Table 3 were aimed at enhancing both breast cancer and cervical cancer screening uptake and reflect the most complete set of cancer screening intervention strategies adopted in practice. In addition to the strategies listed above, strategies aimed at increasing both breast and cervical cancer screening also included screening follow-up, financial incentives, free/subsidized screening services, female physicians, free transportation, and the availability of interpreters.

Only eighteen of the included studies (see Table 4) reported effectiveness based on completion of mammograms or Pap smear, either by self-report and/or verified through clinical record. Of the studies reporting these outcome measures, 8 are randomized control trials (including cluster randomized and randomized controlled crossover trials); 9 are non-equivalent control group designs, and 1 is a prospective cohort study. As reported in Table 5, the Jadad scores of the RCTs are either 2 or 3, indicating that the RCTs included in this review, while not low quality, cannot be classified as being of the highest quality and are therefore subject to some degree of bias.

**Table 4:** Breast and cervical cancer intervention studies: evidence on effectiveness

Study*	**Study Design & Sample Size**	**Description of Intervention**	**Outcomes (Post-Intervention screening rate, Intervention vs. Control)**	**Recommendations**
Thailand 2006 [43]	Non-Equivalent Control Group	An in-home visit by one of the researchers and provided culturally sensitive health education and invitation for cervical cancer screening	Self-reported Pap test: 43.6 vs. 34.9% (p = 0.119)	No significant evidence to support the effectiveness of home visit and invitation.
	304 Thai women in Khon Kaen			
Pennsylvania 2004 [45]	Non-Equivalent Control Group	Participants received cervical cancereducation and patient navigation provided by bilingual Korean health educators.	Self-reported and verified Pap test: 82.7% vs. 22.0% (p < 0.001)	A combination of providing assistance in scheduling/attending screening, community based group education, and culturally sensitive audiovisual materials was recommended.
	102 Korean American women			
Lay Health-Worker Outreach 2001-2004[49-51]	Non-Equivalent Control Groups	Lay health worker outreach (LHWO) that includes small group gatherings, outreach materials, and questions and answers, as well as media education campaign (ME) vs. media education campaign only.	2003 Evaluation results Self-reported Pap test: LHWO + ME from 62.1% to 76.9% (p < 0.001);ME from 70.2 to 72.8% (p < 0.001). 2006 evaluation resultsLHWO + ME: from 65.8 to 81.8% (p < 0.001); ME: from 70.1 to 75.5% (p < 0.001)	Combining the approach of Lay health workers and media education campaign was more effective than media education campaign alone.
	400 (2003 evaluation); 968 (2006 evaluation) Vietnamese Americans			
Seattle 2000-2001[72]	Cluster Randomized Trial	Home visits by outreach workers and invited to group meetings in neighborhood settings.	Self-reported Pap test: increased from 44% to 61% in the intervention group and from 51% to 62% in the control. No significant difference in the increase of the odds of having a Pap test in the two groups	No evidence to support the effectiveness of home visits by outreach workers.
	370 Cambodian American in Seattle, US			
Seattle and Vancouver Trial 1999[52,53]	Randomized Controlled Trial	Outreach intervention which involves tailored counseling and logistic assistance during home visits by trilingual, bicultural outreach workers vs. direct mail intervention vs. no intervention (control)	Self-reported Pap test: Outreach intervention group 39%; direct mail intervention: 25%; control group 15%. The cost effectiveness (cost per additional woman obtaining a Pap test) is less ($304.42) in the outreach arm as compared with direct mail ($485.40).	Outreach intervention which involves tailored counseling and logistic assistance during home visits by trilingual, bicultural outreach workers was found to be more cost effective than direct mail intervention.
	482 (2002 evaluation);139 (2007 cost-effectiveness evaluation) Chinese women in Seattle, Washington, US; and Vancouver, British Columbia, Canada			
Taiwan 1999[54,55]	Non-Equivalent Control Group	Direct-mail campaigns of cervical cancer screening and a phone counseling (intervention) vs. monthly newsletter (control).	Self-reported Pap test: 50% vs. 32% (p = 0.002)	Intervention targeting individual such as direct mail campaigns and phone counseling was found to be more effective than monthly newsletter intervention.
	424 Chinese women in Taiwan.			
Los Angeles 1998-2000[62]	Cluster Randomized Trial	Group sessions conducted at community based organizations, churches, or private homes with some of their peers and a female Filipino health educator; women within each site randomized to receive a cancer screening module (intervention) or a physical activity module (control).	Self-reported mammogram:59% vs. 57% (p = 0.7);Reported Pap test: 56% vs. 52% (p = 0.4)	Small group discussion intervention with health professional was not found to be effective.
	530 Filipino American in Los Angeles, US			
Los Angeles 1998-2000 [36]	Cluster Randomized Trial	An on-site multi-component educational program with on-site mobile mammography at community-based sites where older women gather (intervention) vs. health education only (control).	Self-reported Mammography: 70% vs. 35% (p = 0.015)	The combination of on-site mobile mammography health and education was more effective than health education only.
	499 Older women that include Asian Americans (10% of sample) Women who could not speak English or Spanish were excluded.			
Taiwan 1997-98[56]	Randomized controlled trial	Group teaching program in the workplace on married women’s knowledge, health beliefs and behavior regarding cervical cancer screening (intervention group) vs. pamphlet by mail (control group).	Self-reported Pap test: 90.9% vs. 77.5% (p < 0.05)	Group teaching program in workplace was found to be more effective than pamphlet by mail.
	333 Chinese women in Taiwan: 66(pre-tested experimental group; 57(pretested control group); 64 (nonpretested experimental group; 63(nonpretested control group)			
Alameda 1996-1998[37]	Non Equivalent Control Group – counties 384(intervention); 404(control) Vietnamese Americans in Alameda country, California	Neighborhood-based intervention which involved establishing a Vietnamese Women’s Center in a storefront. Education activities include dissemination of health education materials on breast cancer screening, media campaign, and screening education for Vietnamese physicians.	Self-reported mammogram: 69.6% vs. 58.8%; Reported pap test: 66.9% vs. 65.1%. None of the between-group differences of the differences was statistically significant.	County-wide neighborhood-based intervention involving education, media campaign and screening education for Vietnamese physicians was not found to be effective.
Newham 1995 [38]	Cluster Randomized Trial 2,046 women in Newham, UK: including Indian 348; Pakistani 204; Bangladeshi 123; Chinese 20	A two hour training programme for general practice reception staff	Increase in mammographic screening attendance in general: 9% vs. 4% (p = 0.04); Indian population: 19% vs. 5% (p = 0.005)) Cost: 13 pounds per additional woman screened.	Cultural awareness training for health care professionals was recommended.
Singapore 1994-1996[39]	Randomized Controlled Trial 1500 women in Singapore: Chinese (72.3%); Malays (17.8%); Indians (9.0%)	A routine one-page second reminder letter (R) vs. reminder letter and health education booklet (RP) vs. home visit by a female field worker delivering invitation letter and educational folder (RV)	Mammography attendance: R 7%; RP 7.6%; RV 13.3%. RV vs. R: RR = 1.90 (95% CI 1.27 to 2.84); RV vs. RP: RR = 1.75 (1.19 to 2.59); R vs. RP: RR = 1.09 (0.70 to 1.70)	Home visit delivering the routine second-reminder letter and health educational booklet was more effective than mailing the routine reminder and/or health education booklet; health education booklet did not increase uptake above what can be achieved by routine letter reminder.
Tell a Friend –Alameda 1994-2002 [66]	Non-Equivalent Control Groups – Intervention and Control communities 818 (1994 survey); 72 (1997 survey); 1084 (2002 survey) Korean Americans in Alameda county, California	Community-based interventions that include: 1. delivery of workshops in Korean American churches and distribution of educational materials; 2. adaptation of the American Cancer Society’s “Tell A Friend” program; 3. financial incentives for screening; 4. health councellors were recruited and trained to help organize the church workshops, link women with regular providers and health insurance, promote health as a priority within their churches; 5. educational workshops; and 6. media campaign	Recorded mammogram: 38% vs. 32% (p = 0.108)	Community-based interventions were not shown to be effective in enhancing breast or cervical cancer screening at the community level.
Lay Health Workers Outreach 1992-1996 [69]	Non-Equivalent Control Group – intervention and control cities Vietnamese Americans: 306 (1992 survey); 373 (1996 survey)	Community based small-group sessions conducted by lay health workers; culturally sensitive print materials; media campaigns.	Self-reported Mammogram: 69% vs. 47% (p = 0.006); Self-reported Pap test: 66% vs. 42% (p = 0.001)	A combination of community based group education, culturally sensitive print materials, and media campaigns was recommended.
Media-Led Education Campaign 1992-1994 [70]	Non-Equivalent Control Groups – Intervention and Control counties. 451 (intervention); 482 (control) Vietnamese Americans in California, US	Media-led community culturally sensitive education campaign for breast and cervical cancer screening	Self-reported mammogram: 67.5% vs. 62.6% (p = 0.260); Self-reported Pap test: 66.5% vs. 58.1% (p = 0.014)	Media-led community culturally sensitive education campaign was not found to be effective.
Oldham 1991 [41]	Randomized Controlled Trial 527 Pakistani and Bangladeshi women in Oldham, UK	Two health workers experienced in working with Asian women give encouragement and explanations about breast screening during a home visit.	Self-reported mammogram: 49% vs. 47% (p = 0.53)	Home visit was not found to be effective.
New Zealand 1987-1988 [60]	Prospective Cohort 737 Indian, Pakistani, Bangladeshi in Leicester, New Zealand	Visited and showed a video on the uptake of smear testing vs. visited and shown a leaflet and fact sheet on cervical cancer screening vs. posted a leaflet and fact sheet vs. no intervention (control)	Laboratory computer recorded pap test: 30% of those who received videos; 26% of those who received leaflets; 11% of those mailed leaflets; and 5% of the control group. (p < 0.001)	Personal visits (with video or leaflets) were found to be effective; written translated materials sent by post were found to be ineffective.
Let’s Talk Between Women 2002 [26]	Non-Equivalent 3 Group Design 147 Korean Americans in South California.	Peer-group educational program and low-cost mammography (Let’s Talk group) vs. access to a low-cost mammography alone (Mobile mammography only) vs. no intervention (control)	Self-reported mammogram in Let’s Talk intervention group: 87% (vs. 47% in control group, p < 0.05%); Mobile mammography-only intervention group: 72% (vs. control group, p < 0.05%)	Peer-group educational program was not shown to increase screening more significantly than only providing low-cost mobile mammography.

**Table 5:** Quality Assessment of Randomized Controlled Trials (using Jadad assessment criteria)

**Jadad Quality Criteria**	**Newham 1995**[38]	**Oldham 1991**[41]	**Taiwan 1997-98**[56]	**Los Angeles 1998-2000**[62]	**Los Angeles 1998-2000**[36]	**Singapore 1994-96**[39]	**Seattle/Vancouver 1999**[52]	**Seattle 2000-2001**[72]
Was the study described as randomized?	1	1	1	1	1	1	1	1
Was the method used to generate randomization described and appropriate?#	NR	NR	1	1	1	1	1	1
Was the study described as double blind?	NR	NR	NR	Interviewer blinded	Assessor blinded	NR	NR	NR
Was the method of double blinding described and appropriate?*	NR	NR	NR	NR	NR	NR	NR	NR
Was there a description of withdrawals and dropouts?	1	1	1	1	1	1	1	1
**Total Jadad Score**	**2**	**2**	**3**	**3**	**3**	**3**	**3**	**3**

*Note: NR = Not Reported; 1 = Yes; 0 = No.

Given that eleven of the eighteen selected studies used multiple and highly diversified intervention strategies, it is impossible to identify or estimate the actual effectiveness of any specific intervention strategy. Instead, we examined whether there were evidences to support the overall effectiveness of the intervention programs, and reported the results by each intervention strategy instead of by individual intervention. It should be highlighted that we could not arrive at a conclusive and generalizable conclusion on effectiveness of any one particular intervention.

### Home visit

A 2006 study found no significant evidence to support the effectiveness of home visits by researchers providing culturally-sensitive health education emphasizing the need for Pap smear screening, and inviting Thai women in Khon Kaen, Thailand to participate in Pap smear testing [43]. Similarly, home visits were not found to be effective among Pakistani and Bangladeshi women in Oldham, UK [41]. However, personal visits (with video or leaflets) were found to be more effective than sending written translated materials by post to enhance cervical cancer screening among Indian, Pakistani, Bangladeshi women in Leicester, New Zealand [60]. In the Singapore 1994–1996 study [39], home visits to deliver routine second-reminder letters and health educational booklets were found to be more effective than mailing routine reminders and/or health education booklets (13.3% vs. 7.6%, 95% CI 1.19 to 2.59).

### Media campaign

A media-led culturally sensitive education campaign was not found to be effective among Vietnamese Americans in California [70]. A county-wide neighborhood-based intervention involving education, media campaigns and screening education for Vietnamese physicians, was also found to be ineffective in increasing screening rates [37].

### Mailed culturally sensitive print materials

Mailed translated education materials were not found to be effective among Indian, Pakistani, Bangladeshi women in Leicester, New Zealand [60]. Comparing the effectiveness of direct-mail cervical cancer screening campaigns followed by a phone counseling intervention versus newsletter alone, Hou and colleagues found that interventions targeting individuals through direct mail and phone counseling were more effective than a monthly newsletter intervention (50% vs. 32%, p = 0.002) [37,54]. Seouw and colleagues found that providing health education booklets did not increase uptake above what could have been achieved through routine letter reminders [60].

### Community- or work-based education

Small group discussions with health professionals were not found to be effective in increasing cancer screening among Filipino Americans in Los Angeles [62]. In the “Tell a Friend” Alameda 1994–2002 study, a wide-range of community-based interventions were not shown to be effective in enhancing breast or cervical cancer screening among Korean Americans at the community level [66]. In the Lay Health Workers Outreach 1992–1996 study, media campaigns and the distribution of culturally sensitive print materials, was supplemented by community-based small group sessions delivered by lay health workers [69]. Both mammography and Pap smear screening rates increased significantly among the Vietnamese Americans in the intervention group. In the Pennsylvania 2004 study, Korean American women in the intervention group received cervical cancer education and patient navigation services from bilingual Korean health educators. There was a significant increase in actual cervical cancer screening rates among this group compared with the control group (82.7% vs. 22.0%, p < 0.001). A combination of assistance in scheduling/attending screening, community based group education, and culturally sensitive audiovisual materials increased screening rates among Korean-American women [45]. A workplace-based group-teaching program in Taiwan focused on married women’s knowledge, health beliefs and behavior regarding cervical cancer screening was shown to be more effective than a pamphlet by mail intervention (90.9% vs. 77.5%, p < 0.05) [56].

### Lay health worker outreach

The Lay Health-Worker Outreach 2001–2004 study found that combining lay health worker outreach with a media education campaign was more effective than a media education campaign alone to promote mammography screening among Vietnamese Americans [49-51]. Taylor and colleagues found outreach interventions, which involved tailored counseling, and logistic assistance during home visits by trilingual, bicultural outreach workers was cost effective compared with direct mail (cost per additional woman obtaining a Pap test $304.42 vs. $485.40) [52,53]. In the Seattle 2000–2001 study, Cambodian American women in the intervention group received home visits by outreach workers and were invited to group meetings in neighborhood settings. The study found similar increase in reported Pap test rates in both intervention and control groups. Although no evidence was found to support the effectiveness of home visits by outreach workers, the reported findings might be a result of spillover effect from the target group to the entire community [72].

### Mobile screening services

The Let’s Talk between Women study compared peer-group education programs, in addition to low-cost mammography with providing access to low-cost mammography alone. The peer-group education program did not increase screening as compared with providing low-cost mobile mammography [26]. In the Los Angeles 1998–2000 study, an intervention involving an on-site multi-component education program and mobile mammography at community-based sites where older women gathered was compared with health education. The results suggested that the combination of on-site mobile mammography and health education was more effective than health education alone (70% vs. 35%, p = 0.015) [36].

### Cultural awareness training for health care professionals

In the Newham 1995 study [38], a two-hour cultural awareness training program was provided to general practice reception staff. It resulted in a significant increase in mammogram screening attendance among Asian women as compared with the control group (9% vs. 4%, p = 0.04) [38].

## Discussion

This systematic review of the 37 studies focusing on Asian women synthesized knowledge on the effectiveness of breast and cervical cancer screening interventions. Of these studies, only 18 studies included valid outcome measures (i.e. self-reported or recorded receipt of mammograms or Pap smear). Our review found that intervention studies varied greatly by study population and geographic area. Therefore we could not arrive at a conclusive and generalizable conclusion on effectiveness of any one particular intervention.

We compared the findings of our review with the Cochrane systematic review reports on the large body of literature on breast and cervical cancer screening intervention in western countries that target the general (i.e. Caucasian) population [73,74]. First of all, there is no novel intervention strategy being employed in programs targeting Asian women. The only differences were in how intervention strategies were delivered: intervention studies targeting Asian women adopted cultural sensitive ways to deliver these strategies (e.g. home visit by trilingual and bicultural outreach workers as in the Seattle and Vancouver 1999 Trail) [52,53].

Second, there were some different patterns found in terms of intervention design and effectiveness. Evidences from the Cochrane report favored five breast cancer screening intervention strategies: invitation letter; mailed educational material; invitation letter plus phone call; phone call; and training activities plus direct reminders for the women [73]. For cervical screening intervention, the most recent Cochrane review only found evidences to support the use of invitation letter, and limited evidence to support the effectiveness of the use of educational materials [74].

Among the eighteen intervention studies with valid outcome measures reviewed in our study, eleven used multiple intervention strategies to target individuals in a specific Asian ethnic group. Evidences were found to support the following strategies in increasing mammography intake among certain Asian ethnic women: onsite mobile mammography (among Asian including Korean women in the US) [26,36]; cultural awareness training for health care professionals (among Indian, Pakistani, and Bangladeshi women in the UK) [38]; reminder letter and health education booklet delivered during a home visit (among Chinese, Malay, and Indian women in Singapore) [39]; community based group education plus culturally sensitive educational materials plus and medical campaigns (among Vietnamese women in the US) [69]. For cervical cancer screening, evidences were found to support the effectiveness of the following intervention strategies: community based group education plus culturally sensitive educational materials plus patient navigation (among Korean women in the US) [45]; group education plus outreach materials plus media campaign (among Vietnamese women in the US) [49-51]; home visit plus health education plus patient navigation (among Chinese women in the US and Canada) [52,53]; mail campaign plus phone call (among Chinese women in Taiwan) [54,55]; group education (among Chinese women in Taiwan) [56]. Home visit was found to be effective for a group of Indian, Pakistani, and Bangladeshi women in New Zealand [60], yet ineffective for a group of Thai women in Thailand [43].

Clearly, intervention effectiveness appears to vary with ethnic population, methods of program delivery, and study setting. Compared with the literature of screening intervention on general (i.e. Caucasian) population, the patterns of intervention design and results of effectiveness with those observed from the literature targeting general (i.e. Caucasian) population tend to be more heterogeneous. This is in line with the complexity and challenges in intervention targeting ethnic groups. In summary, our findings supports the hypothesis that employing a combination of multiple strategies is more likely to be successful than single interventions when the target population is ethnic Asian women. The effectiveness of community-based or workplace-based group education programs increases when additional supports, such as assistance in scheduling/attending screening and mobile screening services are provided. Combining cultural awareness training for health care professionals with outreach workers who can help healthcare professionals overcome language and cultural barriers is likely to improve cancer screening uptake. Home visit, invitation letter, media campaigns, and mailed culturally sensitive print materials alone may be ineffective in increasing screening uptake.

Identifying Asian populations to participate in breast and cervical cancer screening studies is challenging. Cultural taboos regarding discussing sexual related topics, and limited enthusiasm for research makes it challenging to recruit Asian women to participate in studies focused on breast and cervical cancer screening uptake [75-83]. Therefore, the validity of findings from small and/or convenient samples is questionable due to uncertainty of selection bias, incomparability between intervention and comparison groups, and lack of statistical power to determine significance.

Most studies used self reports of screening uptake to measure outcomes. Beside recall bias, self-report may be subject to other biases particularly in the case of Asian women where cultural tendencies towards downplaying one’s own opinion and the desire to please others may influence results. These cultural features might lead Asian women to over report screening uptake to demonstrate ‘politeness’ and please research staff [84,85].

In some cases, the level of intervention program exposure was difficult to determine. For example, in the community based studies (such as media and advertisement), it remains questionable whether or not these interventions reached the most hard-to-reach groups.

Finally, only two studies [38,53,72] reported cost information. Economic evaluation of the cost-effectiveness of most intervention studies is essentially infeasible.

## Limitations

Our review had some limitations. First, although we did search bibliographic databases that indexed non-English content, we were not able to search purely non-English databases and so may have missed capturing studies published in smaller journals not indexed in traditional sources. Second, we did not search non-English websites for grey literature and may have overlooked additional content of potential value. Third, the inclusion of both multiple interventions and study designs made infeasible the undertaking of a meta-analysis to quantitatively investigate the effectiveness of any one intervention. Fourth, a majority of the interventions were conducted among Asian Americans (12 out of 18 studies). Cultural differences, health care system especially issues related with access to breast and cervical cancer screening, and other factors specific to the US could confound the results. Caution is needed to generalize and apply the results of this review to particular healthcare systems and settings. Fifth, for Asian immigrants, factors such as number of years since immigration and how well they have adapted in the new culture could have an important impact on their screening results. Unfortunately information is not available uniformly in all selected studies to allow us to assess the potential impacts of acculturation on screening behavior and the effectiveness of interventions. Finally, although both randomized control trials and observational studies were included in our review, we were only able to assess the quality of randomized control trials.

## Conclusions

Promoting breast and cervical cancer screening uptake among Asian women is an important issue for health policy makers. Our systematic review describes the various intervention strategies that have been employed in existing programs. When adopting an intervention program, it is important to consider the impacts of social-and cultural factors specific to the Asian population on cancer screening uptake. Selected studies with valid outcome measures provided evidence on the effectiveness of some interventions. Our review highlighted that such effectiveness may hinge on a variety of factors, such as type of intervention and study population characteristics. While interpretation of results or adopting certain intervention, the large cultural diversities within Asians women should be considered.

Our review identified several issues in the existing literature. First, good quality breast and cervical cancer screening intervention studies on Asian women are still quite limited. Second, a majority of published intervention studies have been conducted among Asian Americans. Third, while some studies demonstrated the effectiveness of certain intervention programs, the cost effectiveness and long-term sustainability of these programs remain questionable. Vigorous study design and economic evaluation methodologies should be employed in future studies to generate valid evidence on the cost-effectiveness of intervention programs. Finally, research is needed to better understand the causal pathways through which these interventions work, as well as the challenges and barriers in implementation. Future research should also focus on developing new and innovative cancer screening interventions and tools to increase cancer screening among Asian women to promote population health and health equity.

## Competing interests

The authors declare that they have no competing interests.

## Authors’ contributions

ML designed the study, conducted the article review, interpreted the results, and drafted and finalized the manuscript. SM participated in the study designed, conducted the article review and revised the manuscript. DL participated in the study design, searched for relevant publications, drafted the Methods section of the text as well as the Tables and revised the manuscript. LS conducted the literature review, prepared the Tables, and revised the manuscript. SS participated in the study design, conducted the article review, and revised the manuscript. HQ proposed the concept, designed the study, conducted the article review and revised the manuscript. All authors read and approved the final manuscript.

## Pre-publication history

The pre-publication history for this paper can be accessed here:

http://www.biomedcentral.com/1471-2458/12/413/prepub
